# Unveiling the potential anti‐cancer activity of calycosin against multivarious cancers with molecular insights: A promising frontier in cancer research

**DOI:** 10.1002/cam4.6924

**Published:** 2024-01-17

**Authors:** Md Sohel, Fatema Tuj Zahra Shova, Shahporan shuvo, Taiyara Mahjabin, Md. Mojnu Mia, Dibyendu Halder, Hafizul Islam, Md Roman Mogal, Partha Biswas, Hasi Rani Saha, Bidhan Chandra Sarkar, Abdullah Al Mamun

**Affiliations:** ^1^ Biochemistry and Molecular Biology Primeasia University Dhaka Bangladesh; ^2^ Biochemistry and Molecular Biology Mawlana Bhashani Science and Technology University Tangail Bangladesh; ^3^ Biotechnology and Genetic Engineering Mawlana Bhashani Science and Technology University Tangail Bangladesh; ^4^ Department of Genetic Engineering and Biotechnology, Faculty of Biological Science and Technology Jashore University of Science and Technology (JUST) Jashore Bangladesh

**Keywords:** anti‐cancer perspective, calycosin, molecular pharmacology, nanoformulation, pharmacokinetics, synergistic

## Abstract

**Background:**

Calycosin may be a potential candidate regarding chemotherapeutic agent, because already some studies against multivarious cancer have been made with this natural compound.

**Aim:**

This review elucidated a brief overview of previous studies on calycosin potential effects on various cancers and its potential mechanism of action.

**Methodology:**

Data retrieved by systematic searches of Google Scholar, PubMed, Science Direct, Web of Science, and Scopus by using keywords including calycosin, cancer types, anti‐cancer mechanism, synergistic, and pharmacokinetic and commonly used tools are BioRender, ChemDraw Professional 16.0, and ADMETlab 2.0.

**Results:**

Based on our review, calycosin is available in nature and effective against around 15 different types of cancer. Generally, the anti‐cancer mechanism of this compound is mediated through a variety of processes, including regulation of apoptotic pathways, cell cycle, angiogenesis and metastasis, oncogenes, enzymatic pathways, and signal transduction process. These study conducted in various study models, including in silico, in vitro, preclinical and clinical models. The molecular framework behind the anti‐cancer effect is targeting some oncogenic and therapeutic proteins and multiple signaling cascades. Therapies based on nano‐formulated calycosin may make excellent nanocarriers for the delivery of this compound to targeted tissue as well as particular organ. This natural compound becomes very effective when combined with other natural compounds and some standard drugs. Moreover, proper use of this compound can reverse resistance to existing anti‐cancer drugs through a variety of strategies. Calycosin showed better pharmacokinetic properties with less toxicity in human bodies.

**Conclusion:**

Calycosin exhibits excellent potential as a therapeutic drug against several cancer types and should be consumed until standard chemotherapeutics are available in pharma markets.

## 1 INTRODUCTION

Cancer remains one of the most formidable challenges to human health, with its devastating impact reaching every corner of the globe.^1^ The severity of cancer seems more than any other disease, including antibiotic resistance and the coronavirus disease 2019 (COVID‐19).^2^ According to the National Cancer Institute (NCI), globally, people are affected by multiple forms of cancer.^3^, ^4^ Some conventional therapies include surgical resection, chemo‐radiotherapies, adjuvant chemotherapies, neoadjuvant therapy, hormonal therapies, monoclonal antibodies, immunotherapies, nanomedicines, and small molecular inhibitors are recommended by oncologists.^5^, ^6^ Despite significant advancements in the understanding and treatment of various malignancies, the quest for novel and effective anti‐cancer agents continues unabated due to having some common drawbacks, like bearing some potential side effects and toxicities.^7^ Therefore, introducing natural compounds derived from plants has long been a source of inspiration for drug discovery due to their rich chemical diversity, historical use in traditional medicine, and reliable therapeutic option for treating many forms of human cancer^8^, ^9^, ^10^, ^11^, ^12^ Currently, medicinal plants or their derivatives account for about 70% of the anti‐cancer compounds, thus, playing the lead role in developing anti‐cancer drugs.^13^, ^14^ Because these compounds are safe, less‐toxic, cost‐effective, and readily available sources from villages to cities and underdeveloped to developed countries.^15^ Consequently, having such kind of facilities is also widely used for other therapeutic advantages.^16^, ^17^, ^18^, ^19^, ^20^

Calycosin, a functional phytoestrogen belonging to the isoflavone category, can be found in the root of *Astragalus membranaceus*.^21^ Calycosin has been traditionally tested in herbal medicine for its myriad of health benefits, including anti‐inflammatory,^22^ anti‐microbial,^23^ neuroprotective,^24^ and anti‐diabetic.^25^ These pharmacological activities of calycosin are due to agonist and antagonist properties with multiple receptors.^26^ However, it is in the realm of oncology that this compound is emerging as a promising candidate for novel anti‐cancer therapies. Some experimental studies of calycosin in numerous study models, including in silico, in vitro, preclinical, and clinical trials against multiple cancer types, have been done.^27^ It has gained attention for its potential anti‐cancer activity due to its ability to influence various cellular processes that play a role in cancer development and progression, including cell cycle arrest, apoptosis induction, inhibition of angiogenesis, anti‐inflammatory effects, metastasis suppression, and hormone receptor modulation: inhibition of signaling pathways and showing antioxidant properties, to prevent the onset and spread of cancer. Additionally, calycosin may work with some other natural compounds and conventional chemotherapy drugs to enhance the activity of these drugs and alleviate the resistance profile of existing drugs.^28^ Calycosin's natural availability, creation of nanocarriers to boost bioavailability, pharmacokinetic profile, and low toxicity make it a popular choice for cancer treatment. As far as we know, although some experimental studies have already been conducted, a detailed overview regarding the onco‐therapeutics potential of calycosin with molecular in treating multivarious cancers has not yet been revealed.

Therefore, the present review aims to shed light on the multifaceted anti‐cancer activities of calycosin and explore the underlying molecular mechanisms, thereby emphasizing its potential significance in cancer treatment, which inspire biological researchers to develop new, effective, clinically proved drugs to combat human malignancies.

## 2 SOURCES OF CALYCOSIN

Calycosin is a natural compound belonging to the isoflavone class of phytochemicals. It can be found in various plant sources, primarily within the Fabaceae (legume) family, including *Thermopsis lanceolata*, and *Hedysarum polybotrys*.^29^ This bioactive chemical compound is mainly found in the desiccated root infusion of some medicinal plants, including *Radix astragali (Astragalus propinquus)*,^30^ *Trifolium pratense L. (red clover)*,^31^ *Astragalus falcatus*,^32^ *A. microcephalus*, *T. fabacea A. membranaceus Fisch. (Bunge)*,^33^ *Wisteria brachybotrys, Thermopsis californica, T. californica, T. lanceolata, Bowdichia nitida, Styphnolobium japonicum, Mucuna membranacea, Andira surinamensis, Myroxylon peruiferum, Calycotome villosa, Oxytropis falcata, Megaselia flavescensand, Pycnanthus angolensis*, many others organism.^34^ Although plant species are available, but its amount and therapeutic dose are not properly analyzed. Some of its derivatives are found in nature. For example, an organic compound called calycosin 7‐O‐glucoside can be discovered in *A. mongholicus*., *Maackia amurensis*, and other organisms.^35^ However, purified herbal medicines might be potential source for calycosin and these are going to use in cancer research. Some herbal medicines that contain Calycosin and have been traditionally used in the for several diseases and planned to cancer are summarized at Table 1.

**TABLE 1:** Reported plant sources of calycosin.

Herbal medicine	Botanical name	Used part	Calycosin content	Common uses	Ref
False lupin	*Thermopsis lanceolata*	Flower	Moderate	Immune support, energy, and vitality	
Sweetvetch	*Hedysarum polybotrys*	NA	Moderate	Digestive health, respiratory support	
Radix astragali	*Astragalus propinquus*	Root	Moderate	Immune system support, energy boost	30
Red clover	*Trifolium pratense* L.	Flowering tops	Significant	Menopausal symptoms, hormone balance	31
Kudzu Root	*Pueraria lobata*	Root	Moderate	Alcohol addiction treatment, blood sugar	38
Black Cohosh	*Actaea racemosa*	NA	Moderate	Menopausal symptom relief, mood stabilization	40
White Willow Bark	*Salix alba*	Bark	Moderate	Pain relief, anti‐inflammatory	44
Astragalus Root	*Astragalus membranaceus* Fisch. (Bunge)	Root	Moderate	Immune system support, energy boost	33
Silky Wisteria	*Wisteria brachybotrys*	NA	Minor	NA	34
Goldenbanner	*Thermopsis californica*			Heart health, blood pressure regulation	34
Japanese pagoda tree	*Styphnolobium japonicum*	Root	Moderate	Anxiety relief, anti‐inflammatory	34
NA	*Mucuna membranacea*	Root	Moderate	Blood thinning, wound healing	34

### 2.1 Astragalus (*Astragalus membranaceus*)

This Chinese herb is known for its immune‐boosting properties and contains calycosin. It is often used as a complementary treatment for various types of cancer to support the immune system during cancer therapy.^36^

### 2.2 Red clover (*Trifolium pratense*)

Red clover is a source of calycosin and has been studied for its potential to inhibit the growth of cancer cells, particularly in hormone‐related cancers like breast cancer.^37^

### 2.3 Kudzu (*Pueraria lobata*)

Kudzu root contains calycosin and has been explored for its anti‐cancer properties, especially in breast cancer. It may help inhibit the proliferation of cancer cells.^38^

### 2.4 Ku Shen (*Sophora flavescens*)

Ku Shen is used in traditional Chinese medicine and contains calycosin. It has been studied for its potential in inhibiting the growth of various cancer cells, including those associated with liver cancer.^39^

### 2.5 Black cohosh (*Cimicifuga racemosa*)

Black cohosh contains calycosin and has been investigated for its potential to inhibit the growth of breast cancer cells. It may also help alleviate some cancer treatment‐related symptoms.^40^

### 2.6 Peony (*Paeonia lactiflora*)

Peony root contains calycosin and has been researched for its anti‐inflammatory and potential anti‐cancer properties. It may inhibit the growth of certain cancer cells.^41^

### 2.7 Radix astragali (Huang Qi)

This traditional Chinese medicine herb contains calycosin and is used in complementary cancer treatments to improve immune function and enhance the body's ability to combat cancer.^42^

### 2.8 Licorice (*Glycyrrhiza glabra*)

Licorice root contains calycosin and has been studied for its anti‐inflammatory and potential anti‐cancer effects. It may be used as part of a broader approach to cancer treatment.^43^

### 2.9 White willow bark (*Salix alba*)

White willow bark contains calycosin and has been researched for its anti‐inflammatory properties. It may be used to manage inflammation associated with cancer including human colon and lung cancer cells.^44^

### 2.10 *Ligusticum chuanxiong* (Chuan Xiong)

This traditional Chinese herb contains calycosin and has been explored for its potential in cancer treatment, particularly in combination with other herbs.^45^

## 3 CHEMISTRY OF CALYCOSIN

**FIGURE 1:**
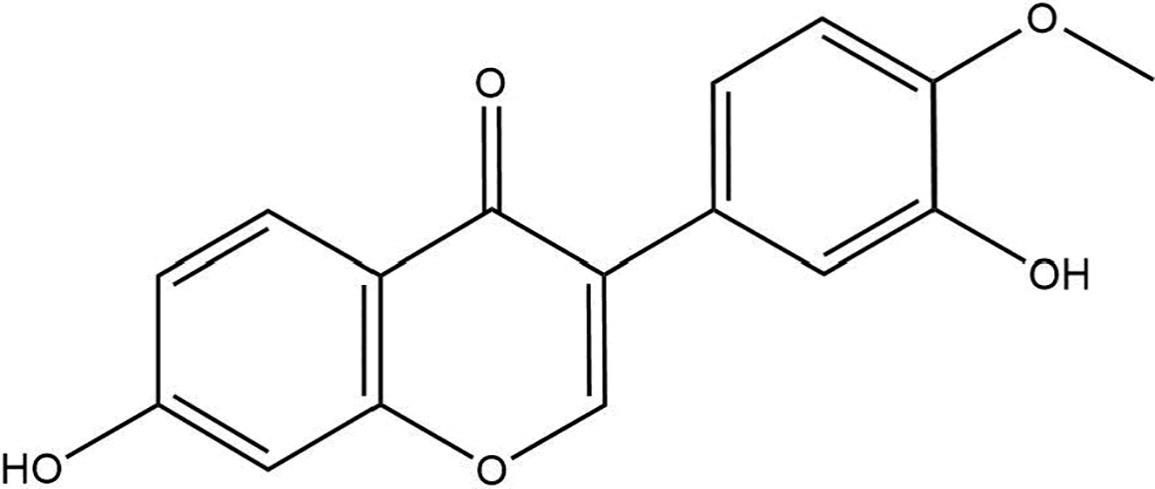
Chemical structure of calycosin.

Calycosin is a purified isoflavone that can conjugate the acid of calycosin (1‐) with the molecular formula C_16_H_12_O_5_, and its IUPAC name is 7‐hydroxy‐3‐(3‐hydroxy‐4‐methoxyphenyl)‐chromen‐4‐one. Calycosin belongs to the family of 7‐hydroxy isoflavones, in which an extra hydroxy group replaces the 3′ position and a methoxy group the 4′ position. It is a member of 7‐hydroxy isoflavones and a member of 4′‐methoxyisoflavones. It has a topological polar surface area of 76 Å^2^, 21 heavy element counts, one chemically bound unit, and no formal charge. The molecular weight of calycosin is 284.26 g/mol, having a white to off‐white powder appearance. The chemical structure of calycosin is depicted at Figure 1.

## 4 THE EXTRACTION, PURIFICATION, AND QUALITY INSPECTION METHODS OF CALYCOSIN

The information related to the extraction, purification, and quality inspection methods of calycosin is very rare. The study focuses on efficiently extracting and purifying calycosin, a bioactive substance found in Astragali Radix. Calycosin‐7‐glucoside can degrade to calycosin. The optimal extraction conditions for calycosin were determined using single‐factor and orthogonal studies. The requirements involved using 100% ethanol and 2.5 mol/L hydrochloric acid in a solid‐to‐liquid ratio of 1:40, followed by a 2‐h compound extraction. The calycosin was purified using a two‐phase solvent system of n‐hexane, ethyl acetate, ethanol, and water (3:5:3:5, v/v). This process yielded 1.3 mg of calycosin with a purity of 95.8% and a recovery rate of 85.9% from 264.9 mg of crude extraction. Purifying calycosin involves isolating it from the plant material and other impurities. Various analytical methods, such as HPLC, melting points, UV, FTIR, ESI‐MS, ^1^H NMR, and ^13^C NMR spectra, were employed to ascertain the structure of calycosin. Hydrolysis with hydrochloric acid is necessary to convert calycosin glycosides into their active state. HSCCC (high‐speed countercurrent chromatography) achieves a high level of purification. Regarding to quality inspections of calycosin, especially in the context of herbal medicine or dietary supplements, various analytical methods including high‐performance liquid chromatography (HPLC), thin‐layer chromatography (TLC), gas chromatography–mass spectrometry (GC–MS), nuclear magnetic resonance (NMR) spectroscopy, ultraviolet–visible (UV–VIS) spectroscopy, mass spectrometry (MS), elemental analysis, microscopic examination, HPLC fingerprints, quantitative analysis, residue analysis, heavy metal analysis, microbiological testing, and stability testing can be done.^46^

## 5 MOLECULAR PHARMACOLOGY AND BASIS OF ANTI‐CANCER ACTIVITY BY CALYCOSIN

Natural phytochemicals or conventional drugs can target a specific receptor to either increase or reduce a particular cell function.^47^ Regarding managing cancerous tissues, phytochemicals must show specific activities by interacting with receptors. Consequently, Chen et al. reported that calycosin mediates anti‐cancer properties, including inhibiting growth and inducing apoptosis by targeting estrogen receptor beta (ER‐β) in numerous breast cancer cells by suppressing IGF‐1R, combined with the selective modulation of MAPK and phosphatidylinositol 3‐kinase (PI3K)/Akt pathways.^48^ Calycosin suppressed the proliferation of ER‐positive and negative breast cancer cells through acting WDR7‐7‐GPR30 signaling via reducing phosphorylation of therapeutic targeting protein including SRC, EGFR, ERK1/2, and Akt in a dose‐dependent manner.^49^ Calycosin exerted anti‐cancer activity in vitro model IGF‐1R mediated cell proliferation by controlling PI3K/Akt signaling pathways and of calycosin‐mediated apoptosis with inhibiting cell invasion by targeting SIRT1 activator which induces activation of AMPK‐induced inhibition of Akt/mTOR axis in HT29 cell line.^50^ Moreover, this molecule could abolish transforming growth factor ß‐(TGF‐β‐) induced epithelial‐to‐mesenchymal transition via altering the Wnt mechanism.^51^ Calycosin interacts with EGFR and thus behaves as an antagonist, downregulating EGFR at in vitro studies with Mice SKBR3 cells and MCF‐7 cells.^49^ The summary of the molecular interaction of calycosin with numerous receptors leading mediate anti‐cancer activities is tabulated in Table 2.

**TABLE 2:** Molecular interaction of calycosin with numerous receptors leading mediate anti‐cancer activities.

Cancer types	Receptor interaction	Molecular basis	Experimental model	Ref
Breast cancer	ERBETA	**↓**MAPK, PI3K/Akt	MCF‐7, T‐47D, MDA‐231, MDA‐435	48
	GPR30	**↓**Phosphorylation of SRC, EGFR, ERK1/2, and Akt	MDA‐MB‐468, SKBR3, MCF‐7, T47D	49
	Androgen receptor (WDR7‐7)	**↓**Phosphorylation of SRC, EGFR, ERK1/2, and Akt	MDA‐MB‐468, SKBR3, MCF‐7, T47D	49
Colorectal cancer	IGF‐1R	**↓**PI3K/Akt signaling pathways miR‐95 expression	SW480 and LoVo	69
Adenocarcinoma	SIRT1	**↓**Activation of AMPK **↓**Akt/mTOR	HT29	50
Colorectal cancer	TGF‐β	**↓**Wnt mechanism PI3K/Akt signaling	SW480 and LoVo	51

## 6 EVIDENCE OF ANTI‐CANCER PERSPECTIVES REGARDING CALYCOSIN

### 6.1 In silico anti‐cancer activity of calycosin

In silico is an early invention process that entails identifying biomarkers, disease progression, and potential treatments for various illnesses. This procedure offers crucial insights into the molecular processes that create and spread cancerous cells in the event of cancer.^52^ Feng et al. reported that apoptosis is a significant factor in biological cell death, where calycosin upregulates Bax, caspase 3, and cleaved caspase 3 and downregulates the expression of Bcl2, determined by GO and KEGG enrichment analyses.^53^ Huang et al. showed in their study, calycosin is effective against colorectal cancer via making interacting with estrogen receptor 2 (ESR2), ATP‐binding cassette sub‐family G member 2 (ABCG2), breast cancer type 1 susceptibility protein (BRCA1), estrogen receptor 1 (ESR1), cytochrome p450 19A1 (CYP19A1), and epidermal growth factor receptor (EGFR). The molecular effect of this interaction is regulating forkhead box protein A1 (FOXA1), transcription factor network, transcription factor 2 (ATF2), transcription factor network, regulation of telomerase, plasma membrane estrogen receptor signaling, estrogen biosynthesis, androgen receptor, and phosphorylation of repair proteins.^54^ CA028 is a derivative of calycosin, and Feiying et al. found that this derivative has potential anti‐cancer activity.^55^ CA028 exerted anti‐tumor effects by reducing CRC cell growth and enhancing the tumor milieu via targeting some genes, including FYN and MAPK1.^55^ By network pharmacology and structural biology, Pan et al. found that calycosin prevents bisphenol A‐induced osteosarcoma.^56^ Tan J. et al. summarized that they identified around 20 biological processes, a rise in the production of the tumor protein p53 (TP53), caspase‐3 (CASP3), and XIAP was primarily responsible for slowing the development of cancer progression.^57^

### 6.2 In vitro and in vivo anti‐cancer activity of calycosin

#### 6.2.1 Breast cancer (BC)

The second most frequent cancer‐related death among women is breast cancer. Previous scientific studies supported that calycosin may be practical therapeutic activity against breast cancer in different study models. It mediated anti‐carcinogenic activity through restrictions on the proliferation of cells and tumor cell growth via upregulating estrogen receptor beta (ER‐β) and downregulating of 1R insulin‐like growth factor 1 (IGF‐1) in MCF‐7 and T‐47D cell in a dose‐dependent way.^58^ In treating human breast cancer cells, calycosin (0.5–100 μM) diminished Akt phosphorylation and reduced PI3K/Akt pathway activation, which results in the downregulation of HOTAIR expression, leading to control proliferation, invasion, and migration and inhibits apoptosis.^59^ In another study, it has shown the activity of calycosin suppresses some cancerous features like cell viability, migration, and invasion through downregulating Foxp3 (forkhead box P3), VEGF (vascular endothelial growth factor), and MMP‐9 (matrix metalloproteinase‐9) in MCF‐7, T‐47D cell lines for breast cancer.^60^ A high dose of calycosin (150 μM) tends to act against cancer by reducing its capacity to migrate and invade, while at low concentrations (20 μM) has been proven to stop the development of cancerous cells and proliferation with inducing apoptosis.^61^ Moreover, calycosin inhibited the proliferation, invasion, and migration of cancer cells through decreasing MMP‐2, MMP‐9, and CD147 levels through downregulating BATF (basic leucine zipper ATF‐like transcription factor) expression and TGFβ1 (transforming growth factor 1) expression.^62^ In vitro study with a low concentration of calycosin has shown decreasing through induction of apoptosis of Bcl‐2 (B‐cell lymphoma 2) and a decrease in Bax (Bcl‐2‐like protein 4).^63^ Calycosin (100 μM) regulates the expression of ERβ, IGF‐1R, PI3K/Akt, and PARP‐1, inducing apoptosis and control of cell proliferation in multiple breast cancer cells.^48^ Studies conducted in both in vitro with Mice SKBR3 cells and MCF‐7 cells, respectively, have shown upregulation of WDR7‐7 (WD repeat domain 7) and downregulation of SRC (a proto‐oncogene), EGFR (epidermal growth factor receptor), ERK1/2 (extracellular signal‐related kinase 1/2), Akt phosphorylation that result in inhibiting proliferation and growth of cancer cells.^49^ Calycosin (6.25–200 μM) prevents the spread of disease and growth of MCF‐7 cell cancer cells, increasing apoptosis by downregulating Bcl‐2 and activating Bax and RASD1.^64^ Li et al. reported that in ER‐positive breast cancer cells, calycosin inhibits migration and invasion of breast cancer cells with a dose of 150 μM via downregulation of Rab27B (member of the RAS oncogene family), VEGF (vascular endothelial growth factor), and β‐catenin allude anti‐cancer activity of calycosin.^65^

In conclusion, a proper dose of calycosin could inhibit the highly invasive features of breast cancer cells. Further studies might show more potentiality of calycosin against breast cancer and suggest it as a therapeutic drug among cancer patients.

#### 6.2.2 Thyroid cancer (TC)

Malignant cell formation in the tissue of the thyroid gland results in the most common endocrine tumor, that is, thyroid cancer. Calycosin has an inverse effect on thyroid cancer, for example, anti‐inflammatory, antioxidant, and anti‐tumor.^66^ Human papillary thyroid cancer cells (B‐CPAP) and human normal thyroid cells (Nthy‐ori 3‐1) were treated with calycosin (0–100 μM), and the result demonstrated that calycosin suppresses the proliferation of cancer cells (B‐CPAP).^66^ The general pharmacological effects on human papillary thyroid cancer cells that are related to the activation of Sestrin2 (SESN2), upregulated p‐AMPK (phosphorylated AMPK), and inhibits p‐mTOR (phosphorylated mTOR) and p‐p70S6Kinase (ribosomal protein S6 kinase).^66^

Overall, calycosin induced anti‐tumor, anti‐inflammatory, and antioxidant function and decreased cancer cell proliferation while regulating many pathways in thyroid cancer therapy.

#### 6.2.3 Colorectal cancer (CRC)

A recent study on cancer data reveals the projected number of new cases of colorectal cancer in both males and females is increasing sequentially. Researchers are working to develop phytochemicals and plant derivatives anti‐cancer agents to treat colorectal cancer.^67^ Calycosin has been proven via a number of studies to slow the progression of colorectal cancer cell. Lin Zu demonstrated that calycosin could suppress the growth of xenograft mouse tumors by inhibiting the PI3K/Akt signaling pathway and increasing ER‐β using siRNA and PTEN.^68^ Another study led by Zhao X. showed that ER‐β, a mediator of the stimulation of ER‐alpha, IGF‐1R, p‐Akt, and miR‐95 expression, can suppress the proliferation of CRC cells caused by calycosin (0–80 μM) in both in vitro and in vivo model.^69^ Chen J. et al. declared their finding calycosin (0–100 μM) reduced cell proliferation, induced apoptosis, and upregulated of PTEN due to an elevation in ER‐β as evaluated by MMT assay, flow cytometry, transwell invasion test, and mRNA levels by rt‐PCR.^70^ Ht29 cell lines were treated with calycosin for 48 h, where calycosin upregulated caspase‐3, BAX, and SIRT1, leading to activate apoptosis and autophagy.^50^ In another recent study, about calycosin (100 μM) work on IGF‐1 stimulates growth and metastasis in CRC cell lines, where it upregulates BAX/Bcl‐2, caspase 3, 9, β‐catenin, BATF2 via STAT3 was with suppression of FOXM1 through STAT1 & NF‐κβ pathways.^71^

In summary, calycosin can provide anti‐colorectal cancer activities by inducing apoptosis and its powerful mechanism. Furthermore, this phytoestrogen regulates cancer‐related growth factors and receptors, suppressing some signal transduction pathways and cell proliferation.

#### 6.2.4 Cervical cancer (CC)

Cervical cancer is a type of cancer that only affects women. The United States reported 14,100 new cases and 4280 fatalities in 2022.^72^ The dietary compounds or natural products such as quercetin (Q), sulforaphane (SFN), genistein (GEN), and epigallocatechin‐3‐gallate (EGCG) have an effect on cervical cell lines like NO production, expression of redox and NO pathway‐associated enzymes, and apoptosis were examined.^73^ The MTT assay showed that calycosin caused a decrease in the viability of SiHa and CaSki cells in a dose‐dependent manner (0–50 μM, 48 h) through decreasing miR‐375 levels.^74^ Calycosin derivatives, including calycosin‐7‐O‐D‐glucoside CG (2.5–100 g/mL), dramatically decreased the expression of Bcl‐2/Bax, leading to an increased apoptosis process in HeLa cells.^75^ Consequently, calycosin can stop the spread of cervical cancer by inducing apoptosis through several molecular mechanisms.

#### 6.2.5 Colon cancer

Colon cancer is a type of cancer that occurs in the large intestine and is caused by the abnormal growth of cells in the colon. The researchers treated colon cancer HCT‐116 cells with calycosin and unraveled the effects on the proliferation of cells and apoptosis via regulation of ERβ‐mediated miR‐17 and PTEN expression in a dose‐dependent manner.^50^

In summary, calycosin effectively treats colon cancer by controlling signaling pathways and suppressing cell proliferation. It also facilitated apoptosis‐related cell death.

#### 6.2.6 Liver/hepatocellular cancer (L/HCC)

Liver cancer, also known as hepatocellular carcinoma, is a kind of cancer that develops in the liver cells. Calycosin has gained attention for its anti‐cancer properties relating to liver cancer. Calycosin promotes apoptosis by reducing the expression of the anti‐apoptotic protein Bcl‐2 and raising the expression of pro‐apoptotic proteins Bax, caspase‐3, and PARP and stopping the cell cycle in the G0/G1 phase through signaling pathways like MAPK, STAT3, NF‐κB, and AKT at hepatocellular carcinoma (HCC) cell lines.^76^ Dongqing Zhang's et al. reported that calycosin appears to block cell division and decrease the length of the G0/G1 stage, potentially by downregulating the expression of TFDP1 and SKP2 genes in vitro models.^77^ In addition, calycosin may increase the expression of specific proteins, including transgelin 2, pyridoxine 5‐phosphate oxidase, stress‐induced phosphoprotein 1, peroxiredoxin 1, and biliverdin reductase B, that are involved in regulating cell proliferation and apoptosis.^77^

In conclusion, the available evidence suggests considering calycosin as a potential therapy for liver cancer, with multiple mechanisms of action, including anti‐proliferative, pro‐apoptotic, and immunomodulatory effects.

#### 6.2.7 Pancreatic cancer (PC)

PC has a lower incidence but a greater fatality rate than other cancers, such as lung, breast, colorectal, and gastric cancer.^78^ Calycosin inhibits cell proliferation by arresting at the S phase in the MIA PaCa‐2 pancreatic cancer cell line, restoring p53 and increasing apoptosis and autophagy through the downregulation of EMT (epithelial‐mesenchymal transition).^79^ In contrast, it was found that the expression of snail, vimentin, and CD31 was increased in the Balb/C nude mice model, suggesting that calycosin may act as an anti‐cancer therapeutic agent by preventing pancreatic cancer cell proliferation.^79^ According to an in vivo study conducted on male C57BL/6N mice, calycosin (25–50 mg/kg) blocks HMGB1/NF‐β signaling with decreasing production of pro‐inflammatory cytokines and chemokines, notably TNF‐α, IL‐6, IL‐1, CXCL‐1, and HMGB1.^80^ Calycosin (25–200 μM) prevented the growth and viability of pancreatic cancer cells (PaCa‐2, PANC1) by triggering p21Waf1/Cip1‐induced cell cycle arrest at S phase and caspase‐dependent apoptosis via caspase‐3,8,9 activation, elevating Bax expression while lowering Bcl‐2, consequently increasing the ratio of Bcl‐2 to Bax.^81^ Calycosin (100 μM) diminished cyclin A1 and the related cyclin‐dependent kinase CDK2 expression while increasing Snail, MMP‐2, ‐9, and p21 gene expression and regulated Raf/MEK/ERK pathway in the pancreatic cell line MIA PaCa‐2.^81^ Calycosin regulated MUC1, Atg5 protein expression snail protein, G1 phase arrest, and TGF‐β in MIA‐PaCa2 cell line at 50–100 μM, leading to cause apoptosis and autophagy, while at 30 mg/kg prevent tumor growth in C57/BL6 mice.^82^ Additionally, in Balb/C mice, calycosin treatment (25–50 mg/kg) hindered NF‐Bҡ/p65 expression, phosphorylation of an IκBα and p38 MAPK, and decreased the level of tumor necrosis factor TNF‐α, IL‐6, and IL‐1. Besides this, it suppressed MPO activity, improved SOD (superoxide dismutase) activity, and caused apoptosis in mice with acute pancreatitis.^83^

Largely, calycosin can limit cell growth and induce apoptosis via activating the caspase pathway, both of which have anti‐pancreatic cancer properties. Additionally, this phytoestrogen controls growth factors and receptors linked to cancer while also inhibiting some signal transduction pathways.

#### 6.2.8 Glioblastoma multiform (GMB)

The major and lethal malignant brain tumor in adults is glioblastoma (GBM), consisting of 16% of all primary brain and central nervous system tumors. It is challenging to diagnose and treat because most drugs are not permeable to the blood–brain barrier. However, in recent years, calycosin has been used to treat glioblastoma effectively. Calycosin (50–800 μM) in U251 and U87 cells can downregulate c‐Met, MMP9, Dtk, Lyn, and PYK2 and p‐AKT expression, resulting in suppression of cell proliferation, invasion, and induce cell death.^27^ In further studies, calycosin inhibits glioblastoma cell proliferation and invasivion, migratory properties by decreasing downstream inflammatory cytokines, including NLRP3, NF‐B, and IL‐1 in glioma cell lines U251, U87, and BALB/c mice.^84^ Calycosin (0–800 mM) downregulates the TGF‐β leading to the inactivation of EMT, MMP‐2, and MMP‐9 and activating N‐cadherin, Snail, and Vimentin in U87 and U251 cell lines.^85^ In another study, calycosin reduced cell viability in U87 cells by exerting antioxidant effect through the Wnt/GSK3−/catenin signaling pathway, regulating SOD, CAT, and GSH levels, induced apoptosis by activating the mitochondria‐associated caspase pathway and regulating pGSK‐3βser9, catenin, and c‐Myc at 1–100 μg/mL.^86^ In treating C6 malignant glioma, the intricate formononetin and calycosin (FMN/CAL) components showed a chemo‐sensitization impact on temozolomide.^87^ Co‐administration of the three drugs may reduce cell growth and increase apoptosis by increasing the expression of these apoptotic proteins: Bax, Cleaved Caspase‐3, Cleaved Caspase‐9, and downregulating anti‐apoptotic protein Bcl‐2 at dose‐dependent manner.^88^

In short, naturally occurring calycosin inhibits the growth, multiplication, and invasion of glioblastoma cells by activating certain proteins linked to apoptosis. Co‐administration of medications with others promotes anti‐cancer activity.

#### 6.2.9 Gastric cancer (GC)

According to cancer statistics from the USA for 2022, this cancer had a mortality rate of 11,090 and a new case rate of 26,380.^72^ The use of Chinese compound medicines like QLD contains a significant amount of calycosin and may be able to prevent the migration and invasion of gastric cancer cell lines.^89^ Zhang Y. et al. investigated the effects of calycosin (47 μM) on increased p27 and p21 expression levels and cell cycle at G0/G1 with lowering cyclin D1, cyclin E, and CDK2, CDK4, and CDK6 levels, in gastric cancer.^90^ Li D. et al. reported that calycosin downregulated the elevated NF‐ҡB, p‐NF‐B, DARPP‐32, and STAT3 in vitro model, while it (40–80 mg/kg) reduced integrin 1 in vivo study.^91^ Zhou L. et al. found that cisplatin/5‐FU/ADM paired with calycosin (10 g/mL) suppressed Akt phosphorylation in cell lines SGC7901, BGC823, and NCIN87 from the human stomach.^28^

Thus, calycosin has the possibility as a potential therapeutic agent for gastric cancer through preventing migration and invasion of cancer cell by downregulating growth factor and increasing tumor suppressing proteins expression and cell cycle arrest.

#### 6.2.10 Nasopharyngeal cancer (NPC)

Globally, one of the most prevalent malignant tumors is nasopharyngeal carcinoma (NPC), which is caused due to genetic factors, EB virus infection, external conditions, and other factors.^92^, ^93^ The pharmaceutical drugs to manage NPC are inadequate, especially for patients with advanced condition.^69^, ^94^ A recent investigation in vitro study demonstrates that calycosin effectively inhibits the proliferation of NPC cells by altering the expression of the lncRNA Ewing sarcoma‐associated transcript 1 in Ewing sarcoma cells.^95^ Treating NPC cells with calycosin (20–50 μM) showed a decreased expression of ESWAT1, p‐TAK1, p‐IкB, and p‐c‐Jun and suppression of NPC cells by mediating downstream effectors TRAF6 and TAK‐1.^92^ Calycosin increases tp53 and caspase 8 levels and decreases MAPK14 expression in a dose‐dependent way resulting in the apoptosis of NPC cells and halting proliferation and the growth of tumor cells.^94^

Taken together, calycosin may operate as a potential anti‐cancer agent by encouraging apoptosis and preventing the proliferation and development of NPC cells by targeting intracellular molecules.

#### 6.2.11 Ovarian cancer (OC)

Ovarian cancer is a kind of cancer that begins in the ovaries. Patients with ovarian cancer typically undergo surgery as their first line of treatment, which usually entails a combination of hysterectomy, bilateral salpingo‐oophorectomy, and removal of the omentum, and platinum compounds.^96^, ^97^ Therefore, creating a cutting‐edge treatment for ovarian cancer is urgently needed.^98^ It has been determined that calycosin contains powerful anti‐cancer pharmacological properties and possesses anti‐cancer function against ovarian cancer via numerous mechanisms. Y. Zhou and his colleagues found that calycosin (100 μM) inhibited SK‐0V‐3 cancer cell growth, proliferation and induced apoptosis via upregulating production of cleaved caspase‐3, cleaved caspase‐9, Bax/Bcl‐2 ratio and downregulating expression of Bcl‐2, pro‐caspase‐3, pro‐caspase‐9 signaling.^98^ Calycosin (50 μM) therapy increased cancer cell apoptosis in the SK‐0V‐3 cell line via upregulating caspase‐3 expression and combining Cisplatin and calycosin induced apoptosis via upregulating expression of several signaling molecules such as P53, caspase‐3, caspase‐9, Bax, and Bcl‐2.^99^

In conclusion, calycosin may operate as an anti‐growth and apoptosis‐inducing agent against ovarian cancer SKOV3 cells by activating caspases and Bcl‐2 family proteins and the p53 pathway making it a prospective therapeutic agent for the treatment of ovarian cancer.

#### 6.2.12 Osteosarcoma (OS)

Osteosarcoma (OS) is a cancerous neoplasm of the bone that primarily affects teenagers, children, and young adults. Like other cancer, calycosin possesses anti‐cancer activities against osteosarcoma in various ways. Calycosin induces apoptosis by upregulating lactate dehydrogenase (LDH) content, caspase‐3, and downregulating miR‐223, PCNA, Bcl‐2, PARP, NF‐κB, p65, and IκBα signaling molecules in 143B cell line, where in vivo study of tumor‐xenograft nude mice, it induces apoptosis and reduces tumor cell mass via downregulating signaling components such as miR‐223, NF‐κB, p65, IκBα, Bcl‐2, and PARP; it mainly suppresses the neoplastic miR‐223‐IκBα pathway.^100^ Qiu et al. conducted a study on BALB/c nude mice at various doges (30–120 mg/kg) and found that calycosin reduced tumor weight via downregulating MPP2, IκBα and ECT2, IL‐6 signaling components, simultaneously activate apoptosis, suppressed cell proliferation, metastasis, and cell growth at various doges (60–180 μmol/L) in 143B human cell line.^101^ Tan et al. found that calycosin induced apoptosis and reduced cell proliferation and growth in the U2OS human cell line by upregulated the expression of TP^53^ and CASP3 and downregulated the expression of XIAP.^57^

In conclusion, the current results show that calycosin has pharmacological effects against osteosarcoma (OS) by both in vivo and in vitro inhibiting the neoplastic IκBa/ECT2 pathway and suppressing the neoplastic miR‐223‐IκBα pathway, both of which are connected to triggering death in tumor cells.

#### 6.2.13 Lung cancer (LC)

Although lung cancer is life‐threatening cancer, effective treatments are not available. Some studies depicted the impact of calycosin against lung cancer as it possesses anti‐tumor, neuroprotective, and anti‐inflammatory properties.^30^ LUAD cells (A549 and H1299) were treated with different concentrations of calycosin, which could cause an increased level of cir_0001946, miR‐21 expression, and decreased HIF‐1*α*.^102^ Moreover, calycosin treatment at A549 cells showed a significant anti‐tumor activity through the underline mechanism of repressing MMP‐2 and MMP‐9 expression levels and inhibiting cell proliferation via controlling cell signaling molecules, including phosphorylation of ERK1/2 along with low levels of integrin β1 at the same cell lines.^103^

In conclusion, the expansion and migration of lung cancer cells were inhibited by the pharmacological effect of Cal via E‐cad/TPA/MMP‐2/Annexin V+/PI‐cells signaling axis in a dose‐dependent way.

#### 6.2.14 Leukemia

Leukemia is a type of blood cancer that is characterized by the rapid development of abnormal blood cells. Dongqing Zhang and his colleagues studied the effects of calycosin downregulate expression of Cyclin D1 to arrest cell cycle at G0/G1 phase leading to inhibit cell proliferation at K562 cell lines.^104^

#### 6.2.15 Melanoma

Melanoma, the most dangerous kind of skin cancer caused several reason including, exposed to ultraviolet (UV) radiation from sunshine, tanning beds, or tanning lamps increases our chance of getting the disease.^105^ Calycosin inhibits melanogenesis by reducing the activity of the PKA/CREB and p38 MAPK signaling pathways. It mainly inhibits the tyrosinase activity and downregulates the expression of MITF and TRP‐2 protein.^106^

**FIGURE 2:**
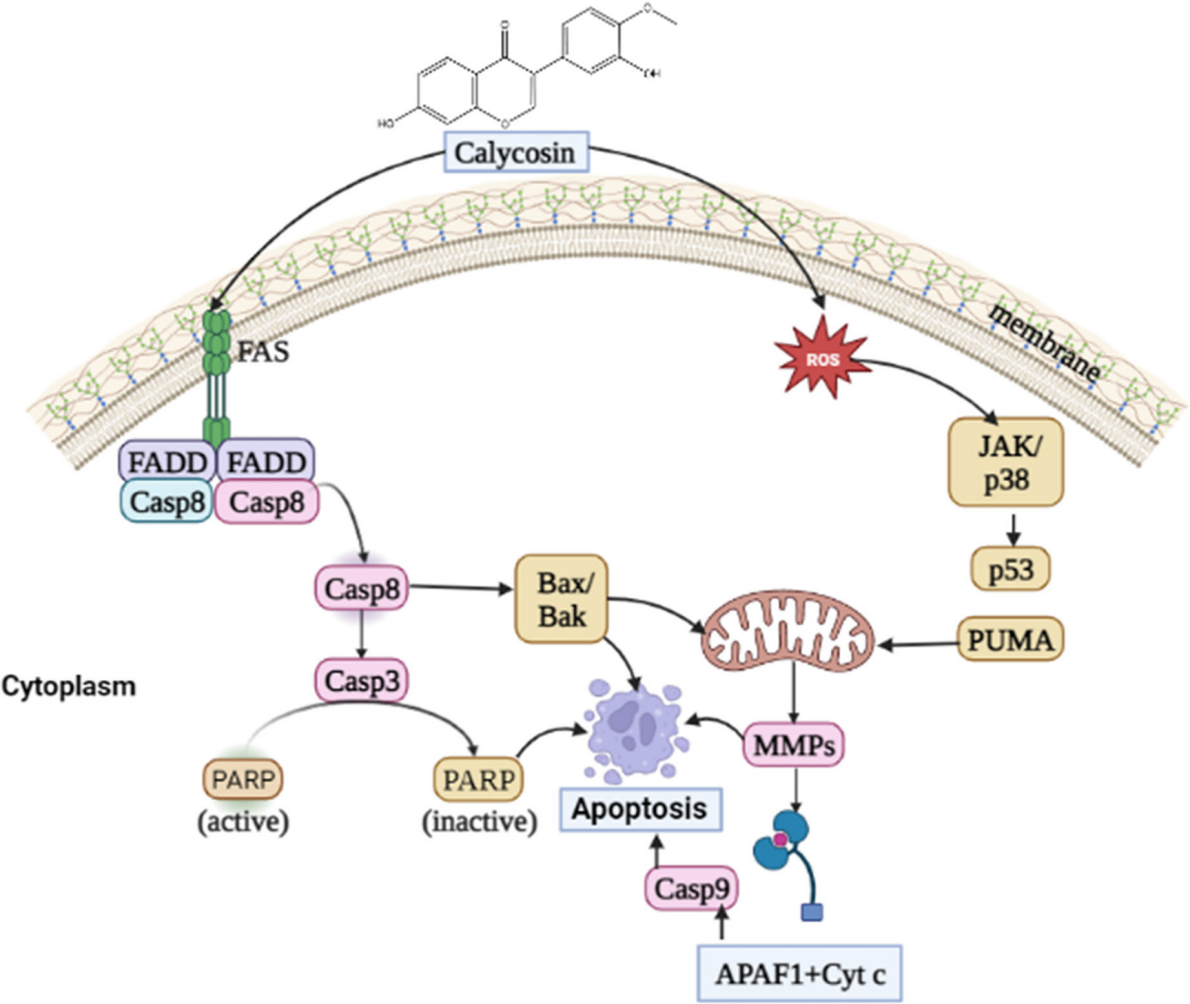
A general mechanism for calycosin‐mediated apoptosis in human malignancies. Calycosin mediates entry through its receptor and activate Caspase 8, inactivate PARP. Furthermore, it increases ROS production and releases Cyt‐c and apaf‐1. All of these combined effects induce death of cancer cell.

**FIGURE 3:**
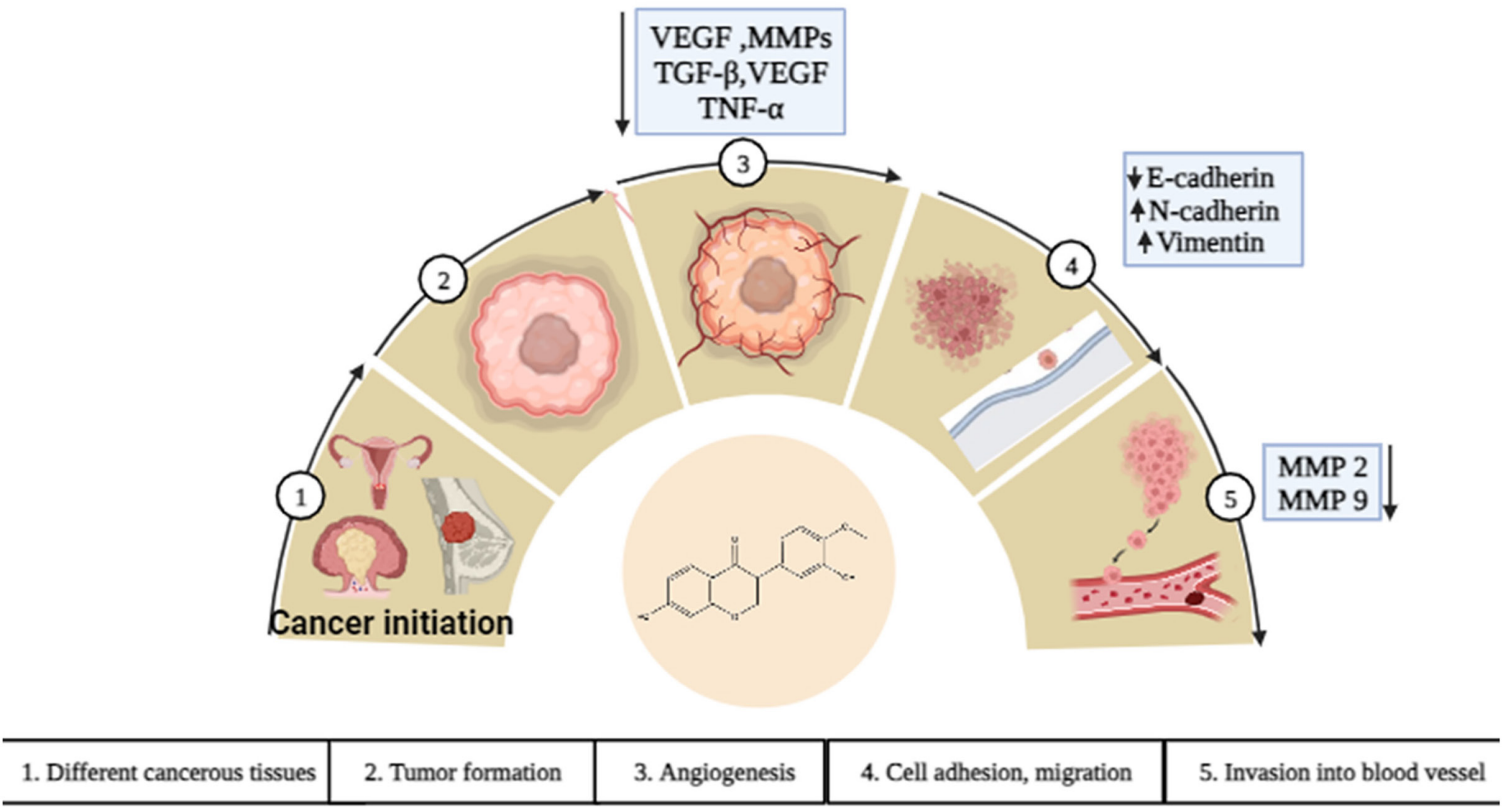
The impact of calycosin on angiogenesis and metastasis in cancer cells. Figure outlines the progression of angiogenesis and metastasis by sequential mechanism. Calycosin interfere at angiogenesis by suppressing angiogenetic factor, including VEGF, MMPs, TGF‐β, VEGF, and TNF‐α, cell adhesion molecules such as E‐cadherin‐cadherin and Vimentin and invasion to blood vessel.

**FIGURE 4:**
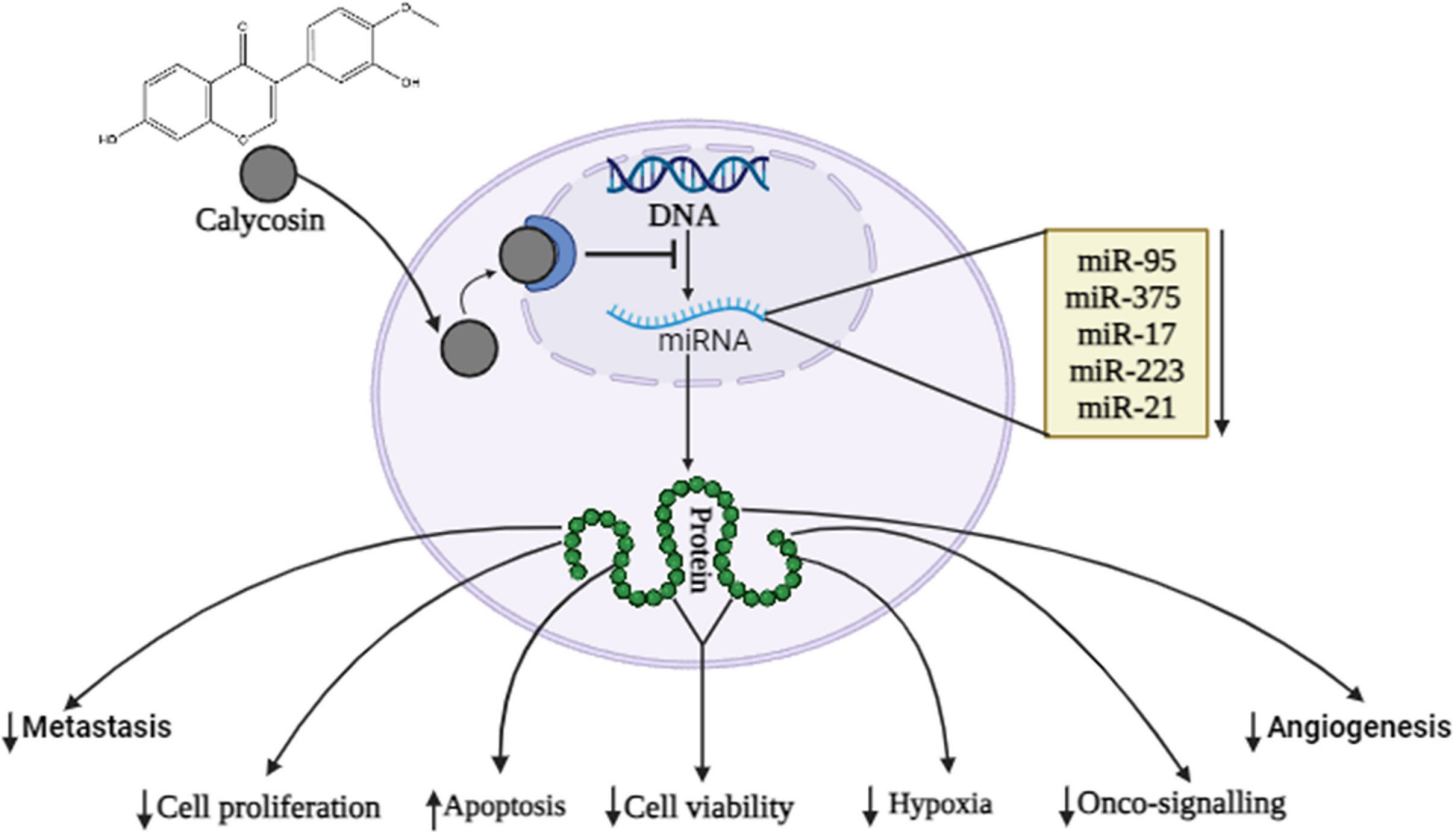
Calycosin modulate miroRNA (miRNA) to fight cancer cells. After interring to the nucleus, it directly alter the transcription and alter miRNA production. This sequence lead to translate some anti‐cancer proteins like apoptosis, cell proliferation, cell viability, hypoxia, and signaling molecules.

Summary of chemotherapeutic activity of calycosin against human malignancies is presented at Table 3. Graphical presentation of anti‐cancer mechanism mediated by calycosin through different mechanism including apoptosis, angiogenesis and metastasis, cell signaling and proliferation and miRNA regulation is presented at Figures 2, 3, 4.

**TABLE 3:** Summary of chemotherapeutic activity of calycosin against human malignancies.

Cancer type	Dose	Type of study (in vitro or in vivo)	Molecular mechanism	Molecular target	Ref
Colorectal	NA	In vitro HCT116, SW480 cell In vivo Mouse xenograft models	↓Cell viability ↑Apoptosis ↓Tumor growth	↑Erβ, PTEN ↓p‐AKT/AKT ratio Bcl‐2 level	68
	0–80 μM	In vitro SW480, LoVo cell In vivo Mouse xenograft models	↓Proliferation ↑Apoptosis	↑Erβ ↓ERα, IGF‐1R, p‐Akt miR‐95	69
	0–100 μM	In vitro HCT‐116 cell	↓Proliferation ↑Apoptosis	↑Erβ, PTEN ↓miR‐17	70
	0–150μM	In vitro LoVo, HCT‐116 cell	↓Proliferation ↑Apoptosis ↓Migration	↑PAI1, BAX, BATF2 ↓PI3K/Akt, STAT3, EMT	51
	50 μM	In vitro HT‐29 cell	↑Cell apoptosis ↓Proliferation, growth, invasion.	↑Becin‐1, LC3II, caspase 3, BAX, SIRT1/AMPK ↓Bcl‐2, p‐SCR, −β1 Integrin, cyclin‐D1, Akt/mTOR	50
		In vitro CRC cell In vivo Nude mice models	↑Cell apoptosis, ↓Proliferation	↑UPS, PP2A, cyt‐c ↓MMP	109
	100 μM	In vitro HCT116, LoVo SW480 cell	↑Cell apoptosis ↓Proliferation, growth, invasion	↑BATF2, caspase‐3, caspase‐9, BAX ↓Bcl‐2, βcatenin, NF‐κβ, MAT2A, FOXM1, IGF‐1	71
Cervical	0–50 μM	In vitro SiHa, CaSki, C‐33A, HeLa Etc1/E6E7 cell	↑Apoptosis, ↓Invasion, Cell viability	↑caspase‐3, LDH, miR‐375	74
	2.5–100μM	In vitro HeLa cells	↑Cell apoptosis	↑BAX, caspase‐3 ↓Bcl‐2	75
Gastric	47 μM	In vitro AGS, KATO‐3, MKN‐28, MKN‐45, NCI‐N87, SNU‐5, SNU‐216, SNU‐484, SNU‐668, YCC‐1, YCC‐6, YCC‐16 cell	↑Cell arrest, apoptosis ↓Migration, metastasis	↑p38, c‐Jun N‐terminal kinase, p21 and p27, ROS ↓SNAI1, E‐cadherin, β‐catenin, CDK2, CDK4, CDK6, cyclin D1, cyclin E, NF‐κβ, STAT3	90
	40–80 mg/kg	In vivo male Sprague–Dawley (SD) rat models	↑Cell apoptosis, ↓Migration, angiogenesis	↓NF‐κβ p‐NF‐κβ, DARPP‐32 and STAT3, β1	91
	10 μg/mL	In vitro SGC7901, BGC823, NCIN87 cell	↑Cell apoptosis ↓Cell invasion, proliferation	↓p‐Akt/Akt	28
Nasopharyngeal	20–50 μM	In vitro CNE1, CNE2 and C666‐1 cell	↓Cell proliferation, ↓Cell growth, ↓Invasiveness	↓EWSAT‐1 ↓TRAF6 ↓TAK‐1	92
	0–50 μM	In vivo CNE1, CNE2 and C666‐1 cell	↓Tumor occurrence and development	↓RNA expression of EWSAT1, TRAF6, TAK‐1	92
	0–80 μM	In vitro CNE1 cell	↓Neoplastic growth, ↓Metastasis, ↑Apoptosis	↑TP53, CASP8, TUNEL‐positive cells, ↓MAPK14	94
Lung	25–100 μM	In vitro A549, BEAS‐2B, H1299 cell	↓Cell colony formation, ↓Invasion, ↓Migration, ↓EMT process	↑circ_0001946, GPD1L ↓HIF‐1*α*	102
	20–40 μM	In vitro A549 Cell	↓Proliferation	↑Annexin V+/PI‐cells	103
	30–40 μM	In vitro A549 cell	↓Cell adhesion, ↓Cell invasion, ↓ECM degradation	↓TPA, MMP‐2, MMP‐9, ERK1/2 signaling pathway, Integrin β1 ↑E‐cadherin	103
Ovarian	100 μM	In vitro SK‐0 V‐3 cell	↑Apoptosis ↓Cell growth ↓Proliferation ↑DNA fragmentation ↓Plasma membrane integrity	↑Bax, Cleaved Caspase‐3Cleaved Caspase‐9 ↑B, ax/Bcl‐2 ratio	98
	50 μM	In vitro SK‐0 V‐3 cell	↑Apoptosis	↑Caspase‐3	99
	NA	In vitro SK‐0 V‐3 cell	↑Apoptosis	↑P53 ↑Caspase‐3, Caspase‐9, Bax	99
Osteosarcoma	30–120 mg/kg	In vivo BALB/c nude mice models	↓Metastasis ↓Tumor weights	↓MPP2 ↓IκBα and ECT2 ↓IL‐6	101
	60–180 μmol/L	In vitro 143B cell	↓Cell growth ↓Proliferation ↑Apoptosis ↓Metastasis	↓PCNA ↓MPP2, ECT2, IκBα	101
	0–40 μM	In vitro U2OS cell	↓Cell proliferation ↑Apoptosis ↓Cell growth	↑TP53 ↑CASP3 ↓XIAP	57
	0–120 mg/kg/day	In vivo Tumor‐xenograft nude mice models	**↓**Tumor mass ↑Apoptosis	↓miR‐223 ↓NF‐κBp65, IκBα ↓Bcl‐2, PARP	100
	60–180 mM	In vitro 143B cell	**↓**Cell Growth ↑Apoptosis ↓Cell proliferation ↓Cell count	↓miR‐223, PCNA, Bcl‐2, PARP ↑Caspase‐3 ↓NF‐Κb, p65, IκBα	100
Breast	0.25–100 μM	In vitro MCF‐7, T‐47D cell	↓Tumor cell growth ↓Cell proliferation ↑Apoptosis	↑ERβ ↓IGF‐1R	58
	0.5–100 μM	In vitro MCF‐7 cell	↓Proliferation ↑Apoptosis ↓Phosphorylation of Akt	↓PI3K/Akt	59
	0.6–150 μM	In vitro MCF‐7, T‐47D cell	↓Cell viability ↓Migration ↓Invasion	↓Foxp3, VEGF, MMP‐9	60
	20 μM	In vitro MCF‐7, T‐47D cell	↓Cancer cell growth ↓Proliferation ↑Apoptosis	↓BRIP1, PARP‐1 cleavage ↑RP11‐65 M17.3, ERα	61
	0–400 μM	In vitro MCF‐7, T‐47D cell	↓Proliferation ↓Migration ↓Invasion	↓BATF, TGFβ1, MMP‐9, MMP‐2 and CD147	62
	200 mM	In vitro MCF‐7 cell	↓Proliferation ↑Apoptosis	↑Bcl‐2 ↓Bax	63
	0–100 μM	In vitro T‐47D cell	↓Proliferation ↑Apoptosis	↑ERβ, PARP‐1 cleavage ↓IGF‐1R, PI3K/Akt	48
	1–32 μM	In vitro MCF‐7 cell In vivo Mice SKBR3 cells	↓Proliferation ↓Cell growth	↑WDR7‐7 ↓SRC, EGFR, ERK1/2, Akt phosphorylation	49
	6.25–200 μM	In vitro MCF‐7 cell	↓Proliferation ↑Apoptosis ↓Cancer cell growth	↓Bcl‐2 ↑Bax ↑RASD1	64
	0–150 μM	In vitro MDA‐MB‐231 cell	↓Migration ↓Invasion	↓Rab27B ↓VEGF ↓β‐catenin	65
Thyroid	0–100 μM	In vitro B‐CPAP, Nthy‐ori 3–1 cell	↓Proliferation ↓Migration	↑SESN2 activation ↑AMPK phosphorylation ↓mTOR	66
Hepatocellular	0.035–1.113 mmol/L	In vitro BEL‐7402, HeLa cell	↓Proliferation ↑Cell apoptosis	↓G0/G1 phase arrest ↓TFDP1, SKP2, CDKN2D ↑CDC2, CDK7, CCNB1 ↑Biliverdin reductase B protein ↓Prx II	77
		In vitro HepG2 cell	↓Proliferation ↑Cell apoptosis ↑Cell cycle arrest	↓Bcl‐2, GF‐β1, SMAD2/3, SLUG ↑Bax, caspase‐3, PARP, MAPK, STAT3, NF‐κB	115
	5 μM	In vitro HepG2 cell	↓Proliferation	↑protein denaturation	113
Pancreatic	N/A	In vitro MIA PaCa‐2 cell In vivo Balb/C nude mice models	↓Cell growth ↑Apoptosis ↑autophagy	↑Arrest at S phase, restoration of p53, Snail, vimentin and CD31 ↓EMT	79
	25–50 mg/kg	In vivo Male C57BL/6N mice models	↑Cell necrosis ↓Tissue development	↑TNF‐α, IL‐6, IL‐1β, CXCL‐1 and HMGB1 ↓HMGB1/NF‐κB signaling, MPO, serum amylase	80
	25–200 μM/30 mg/kg	In vitro MIA PaCa‐2, PANC1 cell In vivo BALB/c mice models	↑Apoptosis ↓Cell growth	↓Raf/MEK/ERK ↑Caspase‐3, ‐8, ‐9, Bax, p21 p21Waf1/Cip1 ↓Bcl‐2, cyclin A1, CDK2, TGF‐β1, PARP	81
	50–100 μM/30 mg/kg	In vitro MIA‐PaCa2 cell In vivo C57/BL6 mice models	↑Apoptosis, ↑Autophagy	↓TGF‐β ↑Atg5, MUC1 gene, snail	82
	25–50 mg/kg	In vivo BALB/c mice models	↑Apoptosis	↓TNF‐α, IL‐6, IL‐1β, MPO, NF‐κB/p65, p38 MAPK, IκBα ↑SOD	83
Glioblastoma	50–800 μM	In vitro HEK293T, U251, U87 cell	↓Proliferation ↑Apoptosis	↓c‐Met, MMP9, P‐AKT, Dtk, Lyn, PYK2	27
	100‐400 μM	In vitro U87, U251 cell In vivo BALB/c mice	↓Cell growth	↓CXCL10, NLRP3, NF‐κB and IL‐1β	84
	0–800 μM	In vitro U87, U251 cell In vivo BALB/c nu/nu mice models	↓Proliferation ↓Migration, ↓Angiogenesis	↓TGF‐β, MMP‐2 and MMP‐9 ↑N‐cadherin, snail, vimentin	85
	1–100 μg/mL	In vitro U87, BV2 cells	↓Cell viability, ↑Apoptosis ↑Cell cycle arrest	↓Bcl‐2, pGSK‐3βser9, β‐catenin, c‐Myc ↑Bax, caspase‐3, ‐9 ↑SOD, CAT, GSH	86
Osteosarcoma	30, 60, 120 mg/kg	In vivo BALB/c nude mice models	↓Metastasis ↓Tumor growth	↓MPP2, IκBα, IL‐6	101
	60, 120, 180 μmol/L	In vitro 143B cell	↓Cell growth ↓Proliferation ↓Metastasis	↓PCNA, MPP2, ECT2, IκBα	101
Leukemia	(0, 30, 60, and 120 mg/kg/day)	In vivo Tumor‐xenograft nude mice models	**↓**Tumor growth ↑Apoptosis	↓miR‐223 ↓NF‐κBp65, IκBα ↓Bcl‐2, PARP	100
	60–180 mM	In vitro 143B cell	**↓**Cell Growth ↑Apoptosis ↓Proliferation	↓miR‐223, PCNA ↓Bcl‐2, PARP ↑Caspase‐3 ↓NF‐Κb, p65, IκBα	100
	20–400 μg/mL	In vitro K562 cell	↓Proliferation ↑Cell growth	↓Cyclin D1 mRNA	104
Melanoma	20–80 μM	In vitro B16F10, HaCaT In vivo Zebrafish embryo models	↑Melanin synthesis, ↓Tyrosinase activity.	↓MITF ↓TRP‐2	106

### 6.3 Clinical trial of calycosin in cancer treatment

Calycosin has been proven to own powerful anti‐cancer properties in silico, in vitro, and in vivo. However, report from a clinical trial of calycosin with cancer is insufficient. A clinical case report conducted by Qiu et al. with osteosarcoma patients reported enhanced levels of neoplastic miR‐223, and elevated expressions of NF‐κBp65, IκBα proteins in tumor cells compared to non‐OS control.^100^ However, 143B cells cell cultures from this patient treated with calycosin downregulate miR‐223 level, Bcl‐2, PARP, NF‐κB, p65, and IκBα, while lactic dehydrogenase (LD) content, and caspase‐3 positive cells were elevated.^100^ Moreover, a controlled randomized trial study conducted by Wang et al. summarized that calycosin accelerated the apoptosis of the LX‐2 cell line but not in cancer cells.^107^

## 7 POTENTIAL SYNERGY OF CALYCOSIN WITH OTHER AGENTS IN CANCER TREATMENT

It has already been established that using natural items to treat cancer has excellent therapeutic potential. These phytochemicals work remarkably well against cancer when combined with standard drugs or other phytochemicals, reducing the adverse effects of many other medications. Regarding glioma treatment, calycosin potentially works in conjunction with temozolomide to decrease tumor density by inhibiting cell migration, inducing cell death, and upregulating apoptotic proteins' expression, including Bax, cleaved caspase‐3, cleaved caspase‐9 and decreased the expression of migratory proteins in the C6 xenograft mouse model.^88^ Calycosin has a good combination with cisplatin, which inhibits the proliferation of laryngeal squamous cell carcinoma and enhances the chemosensitivity of cancer cells to cisplatin.^108^ Moreover, a combination of this compound is effective against gastric cancer cells by preventing protein kinase B phosphorylation (Akt).^28^ Calycosin from Chinese medicine formula (CCMF) exerts synergism via way of the activation of PP2A and inhibition of UPS, activating the mitochondrial apoptosis pathway against multiple cancers, including hepatocellular carcinoma and colorectal cancer.^109^ However, the toxicity and side effects brought on by employing CDDP alone are diminished since its derivative calycosin‐7‐O‐D‐glucoside considerably boosts the CDDP‐induced death of the SK‐OV‐3 cells through the p53 pathway at the cellular level.^99^ Calycosin is also effective with a combination of others phytochemicals. For example, a good combination of formononetin/calycosin can enhance the sensitivity of malignant glioma to TMZ by downregulating NOS2‐dependent cell survival in C6 cells, which provides a basis for the application of this combination in the adjuvant treatment of glioma.^87^ Additionally, Zhang et al. described that a combination of these compounds promoted tumor cells apoptosis through the expression of Bax, cleaved caspase‐3, and cleaved caspase‐9, decreased the expression of Bcl‐2 and inhibiting migration through the expression of matrix metalloproteinase‐2 (MMP‐2) and matrix metalloproteinase‐9 (MMP‐9) in C6 cell.^110^

## 8 POWER OF CALYCOSIN IN ALLEVIATING MULTIDRUG RESISTANCE IN NUMEROUS CANCER TYPES

**FIGURE 5:**
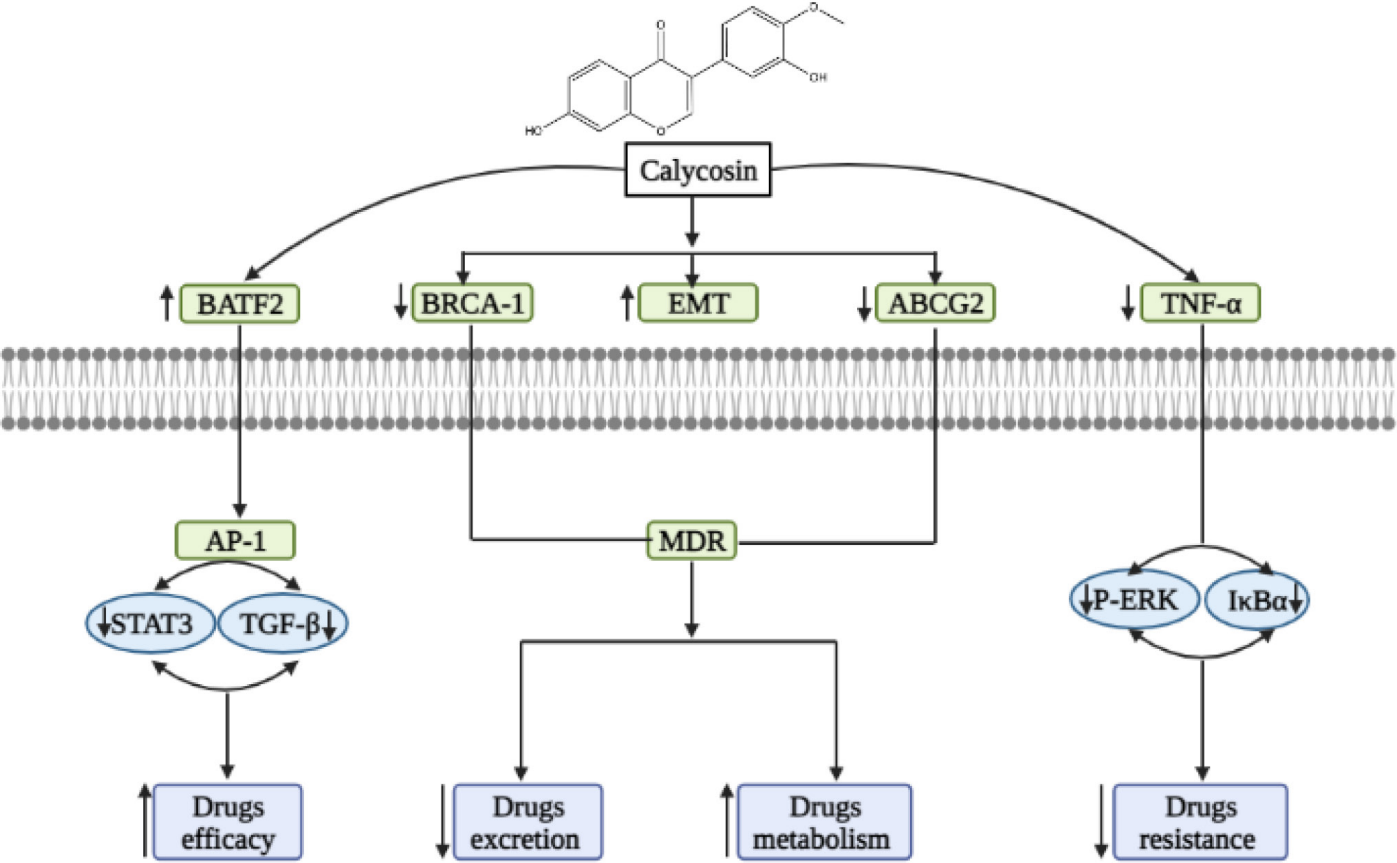
Diagrammatic representation of overcoming multidrug resistance by calycosin via targeting signaling mechanism.

Multidrug resistance (MDR) is a significant obstacle in the fight against cancer. Sometimes anti‐cancer drugs have some level of resistance, which can appear through a variety of mechanisms, such as reduced drug uptake, increased drug efflux, activation of detoxification systems, activation of DNA repair processes and many more.^111^ Many proteins, including P glycoprotein (P‐GP), MRP 1, MRP 1–9, BCRP, and changes in beta‐tubulin, are linked to the occurrence of drug resistance.^112^ Biomedical research is being done on developing chemotherapeutics to overcome MDR in cancer patients. Phytoestrogens like calycosin can be used with other treatments to combat multidrug resistance. Zhou L. et al. discovered that calycosin could increase the suppression of cisplatin to gastric cell lines by preventing protein kinase B from being phosphorylated (Akt). Hence, it is possible to achieve a better therapeutic effect in a lower concentration when cisplatin/5‐FU/ADM is coupled with calycosin.^28^ Epithelial‐to‐mesenchymal transition (EMT) is an important target to overcome drug resistance. Fortunately, calycosin suppresses TGF‐β‐induced epithelial‐to‐mesenchymal transition leading to upregulated expression of BATF2 via the STAT3 pathway by suppressing TGF‐β which controls PCNA expression through the phosphoinositide 3‐kinase pathway in colorectal cancer cells.^51^ IFN‐γ's backbone folded into a more packed structure with increased ‐helix content and a higher melting temperature (Tm), which has an anti‐proliferative impact as a result of the interaction between calycosin and IFN‐γ against HepG2 cells, which are hepatocellular carcinoma, by activating caspase‐9/3.^113^ The combination of calycosin and formononetin decreased the expression of NOS2 in tumor tissues and cells and decreased TNF‐α production in the tumor area.^87^ By stimulating ROS‐mediated MAPK, STAT3, and NF‐B signaling pathways, calycosin inhibited the expression of TGF‐1, SMAD2/3, SLUG, and vimentin and resisted hepatocellular cancer.^76^ Zhu Zhang et al. showed that calycosin caused p21Waf1/Cip1‐induced cell cycle arrest and caspase‐dependent apoptosis while stimulating the migration of MIA PaCa‐2 cells by inducing the epithelial‐mesenchymal transition (EMT) and activating matrix metalloproteinase in pancreatic cancer.^81^ Furthermore, in glioblastoma cell lines, calycosin drastically improve the expressions of mesenchymal‐associated genes/activators, such as N‐cadherin, Snail, and vimentin, and modulated TGF‐β.^85^ Calycosin may have inhibitory effects on ER breast cancer since it at least partially reduces the growth of breast cancer cells through WDR7‐7‐GPR30 signaling.^49^ Moreover, Songya et al. summarized that creating temozolomide as a treatment option for brain tumors with a combination of formonectin/calycosin could improve the sensitivity of C6 malignant glioma to TMZ by preventing NOS2‐dependent cell survival.^87^ In their in silico study, Huang et al. showed that calycosin alters the expression of some multi drugs resistance proteins, including ATP‐binding cassette sub‐family G member 2 (ABCG2) and breast cancer type 1 susceptibility protein (BRCA1) in colorectal cancer.^54^ Overview of synergistic activity and reversing resistance mechanism of calycosin with other phytochemicals and conventional chemotherapeutic agents is shortlisted at Table 4. The graphical presentation of synergistic activity and reversing resistance mechanism of calycosin is summarized at Figure 5.

**TABLE 4:** Overview of synergistic activity of calycosin with other phytochemicals and conventional chemotherapeutic agents.

Cancer type	Combined agents	Cell line	Combined target	Ref
Glioblastoma	Temozolomide	C6 cells	↑Bax, cleaved‐caspases	88
	Formonectin	C6 cells	↓Bcl‐2, MMP‐2 and MMP‐9	110
Laryngeal cancer	Cisplatin	HEp‐2 cell line	↓PI3K‐AKT pathway ↓Erbβ pathway	108
Gastric cancer	Cisplatin 5‐fluorouracil Adriamycin	SGC7901, BGC823 and NCIN87 cells	↓PI3K‐AKT pathway	28

## 9 NANO FORMULATION STRATEGIES OF CALYCOSIN WITH AIMING BETTER BIOAVAILABILITY

Despite having the well‐known therapeutic application of calycosin, it has poor solubility and bioavailability, which can regret its biological activities.^114^ However, this challenge can be solved via nanotechnology because combining phytochemicals with nanocarriers makes them more soluble.^115^ Nanocarriers that have received clinical approval are used to treat and diagnose numerous diseases, including cancer.^116^ diabetic nephrology,^114^ and ischemic heart disease.^117^ Calycosin performed in vitro and in vivo studies with nanostructured lipid carriers against cancer. The surfactants Tween 80, Span 60, PEG 400, and sucrose stearate were administered to the MDA‐MB‐231 cell line before loading calycosin. The drug‐loaded NLCs' average particle size, PDI, zeta potential, spherical shape, 89% drug encapsulation, and 6.5% drug loading capacity were all within the range of 100 nm.^116^ Although calycosin‐nanocarriers related to cancer have limited information, studies with diseases have been done. One of the reasons individuals with acute myocardial infarction experience unsuccessful therapy is insufficient medication. Rats were used in a recent study employing phytochemicals in nano‐systems, and the results indicated the best infarct size reduction and maximum heart accumulation. Mitochondrion‐targeted tetrapeptide (MTP) and cyclic arginyl‐glycyl‐aspartic acid (RGD) peptide co‐modified nano‐system were employed as treatments for co‐loaded calycosin (CAL) and tanshinone (TAN).^118^ For the treatment of diabetic nephropathy, Hung et al. conducted an in vivo and in vitro experiment using free and calycosin‐loaded nanoliposomes. Rats were given an oral dosage of 30 mg/kg; pharmacokinetics activity was improved.^114^ Recent investigations have demonstrated that calycosin can be utilized as a medication, and clinical trials may be carried out using them as the foundation.

## 10 CONTROVERSY AND TOXICITY OF CALYCOSIN

Although calycosin offers many medicinal benefits, the human body may experience only minor adverse effects or harmful responses. Calycosin favors cancer cells when it is present in modest concentrations, including promoting the growth of cancer cells, reducing apoptosis, and downregulating ERK 1 and 2.^61^ In vivo study also showed that calycosin stimulates a dramatic increase in urinary weight.^29^ According to previous research, calycosin promotes angiogenesis in zebrafish larvae and human umbilical vein endothelial cells (HUVEC) both in vitro and in vivo. The zebrafish model also demonstrated that calycosin regulates the ErbB signaling pathway, VEGF (vascular endothelial growth factor), and FGF.^119^ No additional in vivo tests revealed calycosin harm, confirming its efficacy as a treatment.^21^ To determine whether or not calycosin can be harmful by blocking digestive enzymes, its medicinal impact should be thoroughly assessed.^21^

## 11 PHARMACOKINETICS OF CALYCOSIN AND FUTURE PERSPECTIVE IN DRUG DEVELOPMENT

**FIGURE 6:**
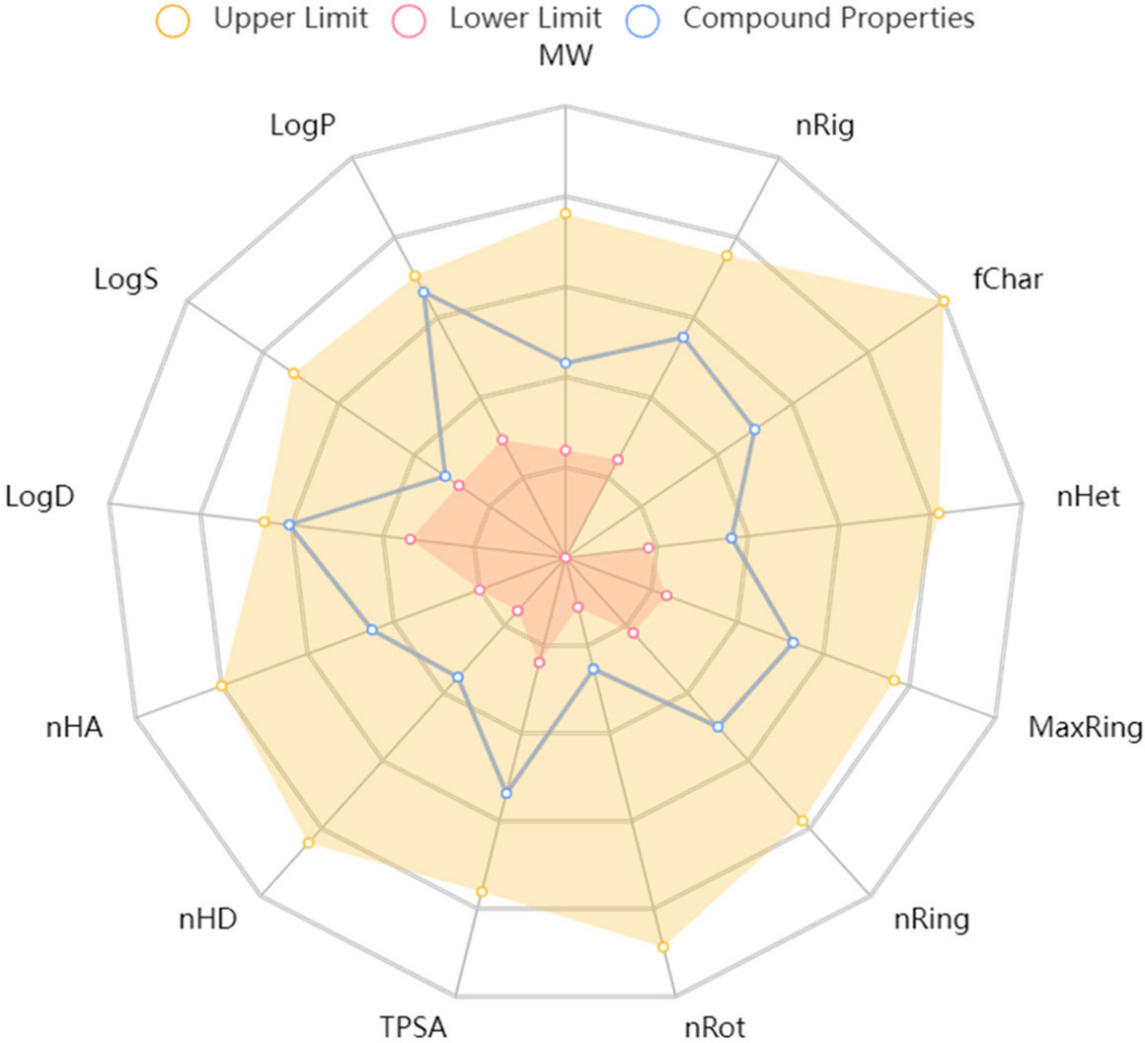
Assessment of physicochemical properties of calycosin.

*In silico* approaches were used to conduct pharmacokinetics or ADME/Tox prediction through computational tools such as Schrodinger's QuickPro modules, online accessible server admetSAR, and SwissADME.^4^, ^120^ It was observed that molecular weight, QPlogPo/w, HBD, and HBA of calycosin were 284.26 g/mol, 1.5, 2, and 5, respectively, indicating good drug‐likeness properties of the compounds. Molecular weight (MW), volume, density, nHA, nHD, nRot, nRing, MaxRing, nHet, fChar, nRig, flexibility, stereo centers, TPSA, logS, logP, and logD were among the physicochemical characteristics radar of calycosin that were computed using the ADMETlab 2.0 software and were shown in Figure 6.

In deciding whether or not to move a substance to the clinical stage, evaluating drug absorption, distribution, metabolism, elimination, and toxicity (ADMET) forecast may help minimize the risk, time, and expense involved. Table 5 summarizes the pharmacokinetic profile of calycosin, predicted using in silico tools like Schrodinger's QuickPro modules and the online server admetSAR, which provides reliable data about absorption, distribution, metabolism, excretion, and toxicity.

**TABLE 5:** In silico drug‐likeness and pharmacokinetics properties profile of calycosin.

Descriptors	Predicted remarks		Comments
	Predicted value	Recommended rage	
Drug likeness			
Molecular weight	284.26 g/mol	130.0–500	Good
QPlogPo/w	1.5	−2.0 to 6.5	Soluble
donorHB	2	0.0–6.0	Good Donor
accptHB	4.8	2.0–20.0	Good Acceptor
Absorption			
Percent human oral absorption	84.75	>80% is high	Highly absorbed
Skin permeability	−3	−8.0 to –1.0	Highly permeable
Caco2 permeability	387.9	25–500	Permeable
MDCK permeability	1.4e‐05	NA	Permeable
P‐glycoprotein substrate	Yes	NA	Effective
Distribution			
BBB permeability	−1.023	−3.0 to 1.2	Good Permeable
CNS permeability	2.2	−2 to +2	Poorly permeable
Human serum albumin	96.8%	60%	High binding affinity
VDs	0.438	NA	Good distribution
Metabolism			
CYP1A2 substrate	Yes	NA/YES	Effective
CYP2C19 substrate	No	NA/YES	Non‐effective
CYP2C9 substrate	Yes	NA/YES	Effective
CYP2D6 substrate	Yes	NA/YES	Effective
CYP3A4 substrate	No	NA/YES	Non‐effective
Toxicity			
Eye irritation	No	NA/YES	Non‐toxic
Hepa‐toxicity	No	NA/YES	Non‐toxic
AMES toxicity	No	NA/YES	Non‐toxic
hERG I inhibitors	No	NA/YES	Non‐toxic
Kidney toxicity	Yes	NA/YES	Toxic
Skin sensitization	Yes	NA/YES	Toxic
Excretion			
CL	7.513		Clearable
T 1/2	0.865		Clearable

Drugs must possess the ability to absorb to reach the bloodstream and be used in daily life. Our predicted result showed that calycosin might be highly absorbed orally and can be permeable by skin, intestine, and kidney cells. Distribution is the movement of a substance within the body and is influenced by several variables, including the permeability of the blood–brain barrier, the permeability of the central nervous system, the binding of the drug to plasma proteins, and the overall volume of distribution. Drugs that attach to plasma proteins (such as human serum albumin, lipoprotein, glycoprotein, and globulins) significantly decrease the amount of the substance in the bloodstream. The fact that our intended chemicals fall within the suggested range suggests that they are likely to move quickly in circulation and reach the target location. The quantity of body fluid needed to disperse in blood plasma is known as the volume of diffusion. Fortunately, calycosin has the probability of distributing uniformly in tissue and blood plasma. In addition, the blood–brain barrier (QPlogBB) and central nervous system are essential for drugs targeting the brain disorder. Unfortunately, calycosin has a poor potential to cross the brain–blood barrier and central nervous system. The CYP450 enzyme detoxifies more than 80% of medications during hepatic first‐pass metabolism, and blocking this enzyme increases drug potency and several adverse effects. The predicted metabolic result reported that calycosin is a substrate for most CYP450 subunits, including CYP1A2, CYP2C9, and CYP2D6. Calycosin's in silico toxicity profile has been predicted based on hepatotoxicity, cardiotoxicity, skin sensitization, kidney toxicity, eye irritation, and AMES toxicity, where calycosin has shown toxicity against ll except AMES toxicity. We used total clearance (CLtot) and T 12 to estimate the clearance profile based on the compounds' kidney clearance characteristics. As anticipated, the findings showed that calycosin can be easily eliminated from the body once it has served its intended medicinal purpose.

Therefore, pharmacokinetics profile of calycosin suggests good lead and efficiency with fewer toxicity risks. So, calycosin can be a good starting source of drug design, such as docking and pharmacophore‐based virtual screening in cancer treatment and drug discovery communities.

## 12 CONCLUSION AND LIMITATIONS OF THIS STUDY

**FIGURE 7:**
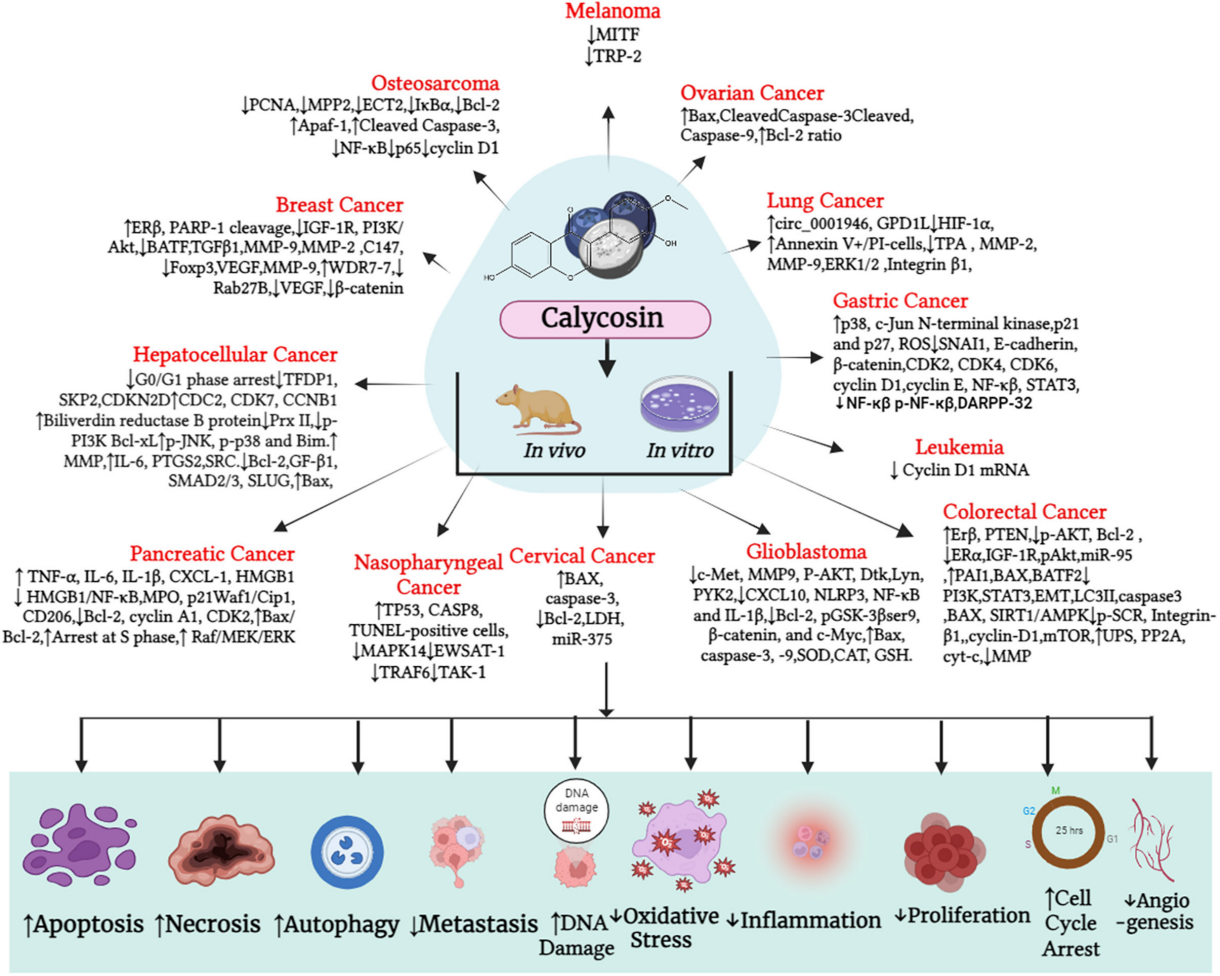
Overall graphical presentation of molecular mechanism of calycosin that mediates anti‐cancer properties.

Based on the evidence presented in this current review, calycosin may be a prospective molecular lead for developing anti‐cancer drugs. As far as we know, this is the first review article with calycosin against numerous cancers. This compound mediated anti‐cancer activities against multiple cancer due to the specificity of some receptors, including estrogen receptor, IGF‐1R, and EGFR. The detail mechanism of anti‐cancer activity from in silico, in vitro, preclinical, and clinical studies suggested that calycosin mediated its anti‐cancer efficacy through targeting apoptosis protein, anti‐apoptotic proteins, among others (Bcl‐2) and apoptotic proteins (Bax, Bak, caspase), arresting cell cycle as well as cell proliferation, including inhibiting expression and activity of cyclins (A1, D1, E) and CDKs (2, 4, 6), or increasing the expression of CDKs inhibitors (p21, p27, p53), metastasis and angiogenesis (MMP‐2& 9, Wnt/‐catenin, PARP), regulating inflammation (TNF‐α, NF‐κB, IκB kinase, IL‐1β, phospho‐Akt, phosphor‐p65), targeting cell signaling (NF‐κB, PI3K/Akt, MAPK/ERK, p‐mTO) in numerous cancer cell lines (Figure 7). Also, it has been shown that calycosin synergistically increases the anti‐cancer activities of other natural products and some conventional drugs after conjugation with them and alleviates the resistance profile of anti‐cancer drugs. Information on calycosin about nanotechnological approaches in cancer treatment is limited, so further studies with calycosin nanotechnology are needed to improve target cancer tissues. Furthermore, having good pharmacokinetics with lesser toxicities suggests that this natural compound may become a promising drug discovery candidate after using other techniques like network pharmacology and molecular docking against many diseases, especially cancer. It's worth mentioning that while these potential mechanisms of anti‐cancer activity are supported by preclinical studies conducted in cell lines and animal models, more research is needed to establish the effectiveness of calycosin in human cancer therapy. Clinical trials are essential to determine its safety, optimal dosage, and actual benefits in treating various types of cancers.

## AUTHOR CONTRIBUTIONS

**Md Sohel:** Conceptualization (lead); resources (equal); software (equal); writing – original draft (equal); writing – review and editing (equal). **Fatema Tuj Zahra Shova:** Writing – original draft (equal). **Shahporan Shuvo:** Writing – original draft (equal). **Taiyara Mahjabin:** Writing – original draft (equal). **Md. Mojnu Mia:** Writing – original draft (equal). **Dibyendu Halder:** Writing – original draft (equal). **Hafizul Islam:** Writing – original draft (equal). **Md Roman Mogal:** Writing – original draft (equal). **Partha Biswas:** Writing – original draft (equal). **Hasi Rani Saha:** Resources (equal); writing – original draft (equal). **Bidhan Chandra Sarkar:** Writing – original draft (equal). **Md. Abdullah Al Mamun:** Conceptualization (equal); writing – review and editing (equal).

## FUNDING INFORMATION

This research did not receive any specific grant from funding agencies in the public, commercial, or not‐for‐profit sectors.

## CONFLICT OF INTEREST STATEMENT

The authors have declared that there are no conflicts of interests.

## Data Availability

Data are included in article/supplementary material/referenced in article.
